# Therapeutic Efficacy of Rapamycin in an Experimental Mouse Model of Corneal Alkali Burn

**DOI:** 10.3390/ijms27083688

**Published:** 2026-04-21

**Authors:** Basanta Bhujel, Hun Lee, Ho Seok Chung, Jae Yong Kim

**Affiliations:** 1 Department of Ophthalmology, Asan Medical Center, College of Medicine, University of Ulsan, Seoul 05505, Republic of Korea; basantabhujel86@gmail.com (B.B.);; 2 Department of Medical Science, Graduate School, University of Ulsan, Seoul 05505, Republic of Korea

**Keywords:** rapamycin, cyclosporine A, alkali burn, cornea, inflammation, wound healing

## Abstract

Corneal alkali burn induces severe inflammation and tissue damage, leading to loss of corneal transparency and vision impairment. In this study, we evaluated the therapeutic potential of rapamycin (RAPA) compared with cyclosporine A (CsA) in a mouse model of corneal alkali burn, focusing on nuclear factor kappa-light-chain-enhancer of activated B cells (NF-κB)–mediated inflammatory signaling and its impact on corneal wound healing and repair. Notably, RAPA robustly suppressed NF-κB activation, reduced infiltration of F4/80 macrophages and MPO neutrophils, and downregulated pro-inflammatory cytokines, including TNF-α, IL-1β, and IL-6. RAPA also markedly inhibited corneal neovascularization, as evidenced by decreased VEGF expression, reduced CD31 vessel formation, and suppression of Ang-2. RAPA substantially inhibited pathological fibrotic remodeling by reducing TGF-β1 expression, attenuating myofibroblast activation (α-SMA), decreasing collagen III deposition, and modulating matrix remodeling through suppression of MMP-9. Crucially, RAPA preserved epithelial barrier integrity by maintaining occludin expression, supported proper epithelial differentiation through sustained expression of CK12, and enhanced mucin layer stability by increasing MUC1 expression. It also restored tear production, reduced apoptotic cell death (TUNEL), and decreased dysregulated epithelial proliferation (Ki67). In conclusion, RAPA showed superior efficacy compared with CsA, primarily by enhancing corneal wound healing and facilitating structural and functional outcomes in the burned cornea. These findings underscore RAPA as a promising therapeutic candidate for ocular surface repair and vision restoration in extensive corneal injury.

## 1. Introduction

Corneal alkali burn represents one of the most severe ocular surface injuries and remains a major cause of vision loss worldwide. The rapid penetration of alkali agents into the cornea results in extensive epithelial and stromal necrosis, leading to persistent epithelial defects, corneal opacity, neovascularization, and ultimately blindness [1].

The pathophysiology of alkali-induced corneal damage is a complex and multifactorial process. Upon exposure, alkali rapidly saponifies cell membranes and disrupts extracellular matrix (ECM) integrity, allowing deep stromal infiltration [2]. This initial insult also destabilizes the tear film, leading to reduced tear volume and quality, as well as marked depletion of membrane-associated and secretory mucins [3]. These structural and functional disruptions trigger acute inflammation characterized by massive recruitment of neutrophils and macrophages, accompanied by excessive release of pro-inflammatory cytokines such as interleukin-1β (IL-1β), tumor necrosis factor-α (TNF-α), interleukin-6 (IL-6), and matrix metalloproteinases (MMPs) [4]. The sustained inflammatory environment induces extensive epithelial apoptosis, disrupts epithelial barrier integrity, and impairs differentiation of epithelial cells, thereby delaying resurfacing of the burned cornea. Concurrently, upregulation of vascular endothelial growth factor (VEGF) promotes pathological angiogenesis, and persistent activation of α-smooth muscle actin (α-SMA) expressing myofibroblasts promotes abnormal collagen deposition, leading to stromal fibrosis and scarring [4]. Together, these processes establish a vicious cycle of inflammation, neovascularization, and fibrosis, impairing corneal clarity and visual recovery [5].

Current therapeutic strategies primarily focus on reducing inflammation and preventing further tissue damage. Conventional approaches include intensive lubrication, topical corticosteroids, antibiotics, and anti-VEGF agents [6]. Corticosteroids are effective in rapidly suppressing inflammation, but their prolonged use may be associated with adverse side effects, such as cataracts, glaucoma, and corneal melting [7]. Despite these available therapies, severe alkali burns frequently result in dysregulated corneal wound-healing progression, characterized by persistent inflammation, pathological neovascularization, stromal fibrosis, and incomplete tissue regeneration, underscoring the need to explore additional agents capable of comprehensively modulating these interconnected processes [3,8].

In this context, Rapamycin (RAPA) has gained increasing attention as a versatile therapeutic agent for ocular surface injuries. As a macrolide compound that potently inhibits the mammalian target of rapamycin (mTOR) pathway, RAPA exerts broad regulatory effects on cellular growth, inflammation, and tissue repair [9]. Originally developed as an antifungal, it is now widely recognized for its immunosuppressive, anti-proliferative, and anti-angiogenic properties [10]. Clinically, it is approved for preventing organ transplant rejection and is being explored in a variety of inflammatory and proliferative disorders [11,12]. Its ability to modulate both innate and adaptive immune responses offers broader therapeutic coverage compared to conventional immunosuppressants [13].

Building on previous preclinical insights into the protective actions of RAPA in ocular surface injury models, the present study aimed to compare the therapeutic efficacy of RAPA and cyclosporine A (CsA) to further evaluate the overall protective potential of RAPA as a follow-up investigation [10,14,15,16]. In this study, 0.05% CsA was selected based on its well-documented immunosuppressive and anti-inflammatory effects in multiple ocular conditions, as reported by a plethora of studies [17,18,19,20]. In contrast, 0.05% RAPA was included as an experimental agent due to its emerging pleiotropic protective effects in ocular surface diseases [21].

While RAPA and CsA have been comparatively studied in corneal allograft transplantation models [22,23], their relative effects in an alkali-induced corneal burn, particularly on nuclear factor kappa-light-chain-enhancer of activated B cells (NF-κB)–mediated inflammatory and downstream pathological responses, remain unclear. We therefore hypothesized that RAPA would enhance overall corneal wound healing following an alkali burn by modulating the NF-κB inflammatory activation pathway, ultimately leading to superior restoration of corneal structure and function in a mouse model. Our study addresses this gap by directly comparing the therapeutic effects of RAPA and CsA in an experimental mouse model of corneal alkali burn to provide a scientific basis for developing improved treatment strategies for severe corneal alkali burn.

## 2. Results

### 2.1. Effects of RAPA on Ocular Surface Integrity

Fluorescein staining was performed to evaluate corneal epithelial defects and overall ocular surface integrity in the healthy, burn, burn + CsA, and burn + RAPA-treated groups. The burn group exhibited intense fluorescein uptake and extensive epithelial disruption, indicating severe corneal injury.

**Figure 1:**
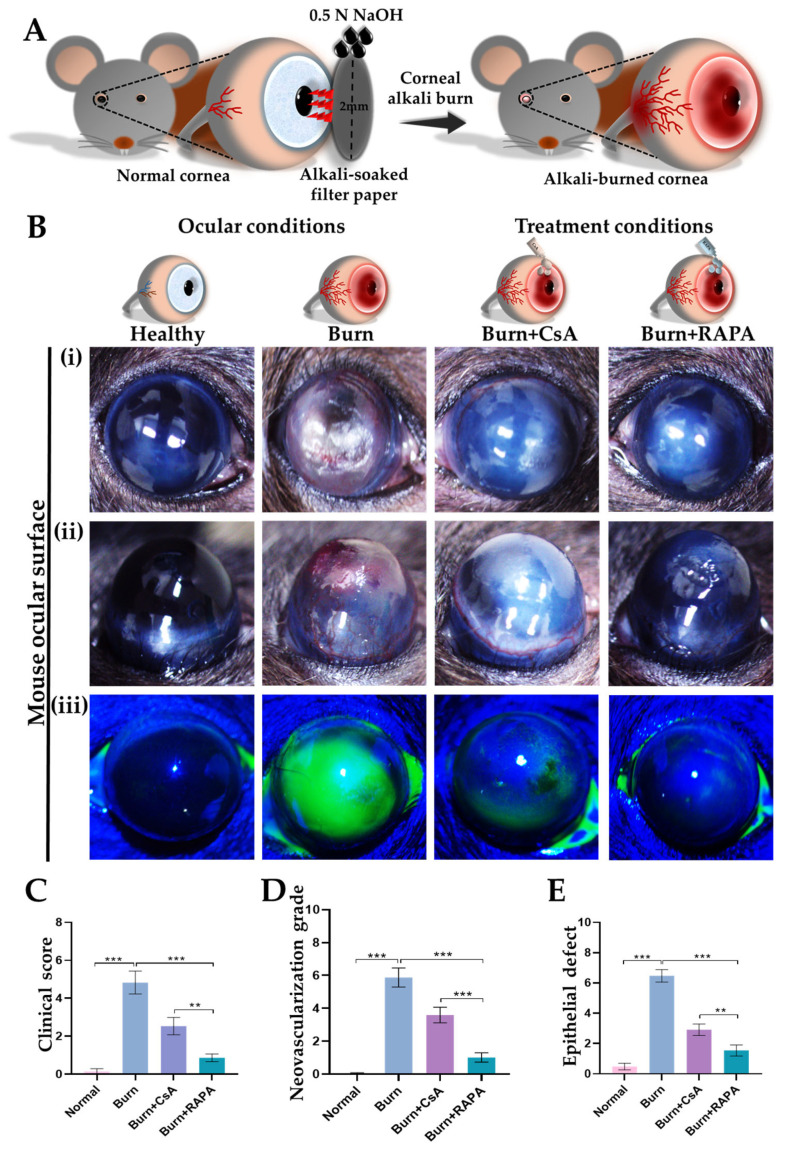
Therapeutic potential of RAPA on the ocular surface in an alkali burn mouse model. (**A**) Schematic illustration of the experimental corneal alkali burn model in mice. A 2 mm filter paper disc soaked in 0.5 N alkali solution is applied to the central cornea for 15 s to induce a corneal alkali burn. The schematic illustrates ocular surface damage, disruption of normal corneal architecture, and the development of corneal neovascularization following an alkali burn. (**B**) (**i**,**ii**) Slit lamp examination. (**iii**) Fluorescein staining. (**C**) Quantitative assessment of clinical score. (**D**) Quantitative analysis of neovascularization grade. (**E**) Quantitative evaluation of epithelial defect area. Scale bar = 1 mm. Mean ± SEM (*n* = 10). One-way ANOVA followed by Tukey’s post hoc test. ** *p*  <  0.01; *** *p*  <  0.001.

Notably, RAPA-treated corneas demonstrated a significantly smaller epithelial defect area than those treated with CsA, indicating a superior effect of RAPA on corneal epithelial wound closure. Healthy corneas, however, exhibited no fluorescein staining (Figure 1B(iii),E).

### 2.2. Effects of RAPA on Corneal Clinical Scores

We further performed corneal clinical scoring to assess the severity of ocular surface damage in the healthy, burn, burn + CsA, and burn + RAPA-treated groups. The burn group exhibited a pronounced clinical score compared with healthy corneas, reflecting severe epithelial disruption, opacity, and inflammation following an alkali burn.

Importantly, RAPA treatment effectively reduced clinical scores compared to the burn group and CsA-treated group, demonstrating comparable protective effects. Clinical scores in healthy control eyes remained unchanged, confirming preserved corneal integrity (Figure 1B(i),C).

### 2.3. Effects of RAPA on Corneal Neovascularization Grade

Next, corneal neovascularization was evaluated to assess pathological angiogenesis following an alkali burn in the healthy, burn, burn + CsA, and burn + RAPA-treated groups. Compared with CsA treatment, RAPA administration resulted in a significantly lower neovascularization grade, indicating a more pronounced anti-angiogenic effect.

In comparison, the untreated burn group exhibited the highest neovascularization grade among all groups, reflecting a robust angiogenic response to burn-induced corneal injury. No vascularization was observed in the healthy group, reflecting normal corneal transparency (Figure 1B(ii),D).

### 2.4. Effects of RAPA on Tear Production and Corneal Surface Mucin Expression

**Figure 2:**
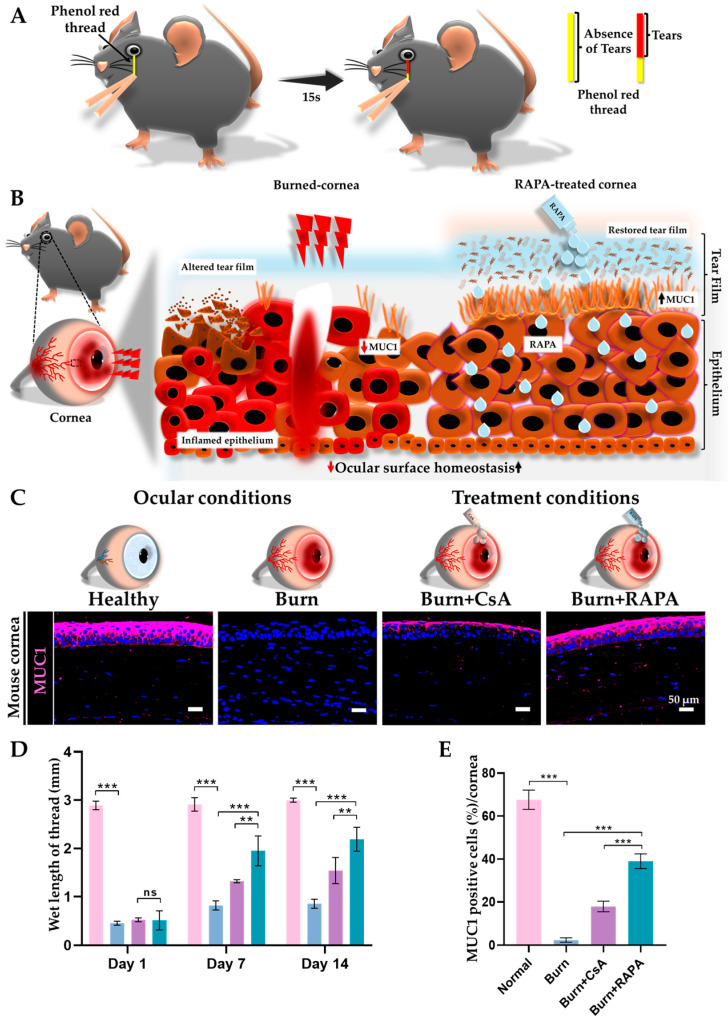
RAPA preserves the ocular surface homeostasis in an alkali burn mouse model. (**A**) Schematic overview of the phenol red thread test for the assessment of tear secretion in mice. A phenol red–impregnated thread, which is yellow under neutral conditions, is placed on the ocular surface for 15 s. Contact with the alkaline tear fluid induces a color change from yellow to red, and the length of the color-changed portion is measured in millimeters (mm) as an indicator of tear secretion. (**B**) Conceptual illustration of tear film and mucin alterations in the cornea following an alkali burn and RAPA treatment. Alkali burn disrupts tear film stability and reduces the protective mucin layer, as indicated by decreased mucin 1 (MUC1) expression in the corneal epithelium. In contrast, RAPA treatment preserves tear film integrity and maintains MUC1 expression, supporting epithelial barrier function and promoting ocular surface homeostasis. (**C**) Immunofluorescence staining for MUC1 in the cornea. Nuclei were counterstained with DAPI for visualization. (**D**) Quantitative assessment of tear measurement. (**E**) Quantification of MUC1-positive cells in the cornea. Immunopositivity in the corneal tissue was quantified relative to the total number of DAPI-positive cells. Mean ± SEM (*n* = 7). One-way ANOVA followed by Tukey’s post hoc test. ** *p*  <  0.01; *** *p*  <  0.001; ns, not significant.

Tear production was assessed to evaluate functional recovery of the ocular surface in the healthy, burn, burn + CsA, and burn + RAPA-treated groups. Tear secretion was significantly reduced in the burn group compared with healthy controls, indicating impaired ocular surface function following an alkali burn. Interestingly, RAPA treatment significantly increased tear production compared with both the burn group and CsA-treated group. The healthy group maintained a baseline tear production, confirming normal ocular surface function (Figure 2D).

Analysis of corneal mucin-1 (MUC1) expression revealed a marked reduction in the burn group, reflecting disruption of the epithelial glycocalyx. Notably, RAPA-treated corneas exhibited more continuous MUC1 expression compared with CsA-treated corneas, suggesting a more effective restoration of the corneal mucin layer. Healthy corneas maintained uniform MUC1 expression across the surface (Figure 2C,E).

### 2.5. Effects of RAPA on Corneal Epithelial Barrier Integrity, Tight Junction Organization, and Differentiation

Corneal epithelial barrier integrity, tight junction organization, and epithelial differentiation were evaluated by immunofluorescence staining for occludin, zonula occludens-1 (ZO-1), and cytokeratin-12 (CK12), respectively, in the healthy, burn, burn + CsA, and burn + RAPA-treated groups. The untreated burn group showed marked disruption of occludin and ZO-1 localization, accompanied by a pronounced reduction in CK12 expression, indicating severe impairment of epithelial barrier function and differentiation following an alkali burn.

**Figure 3:**
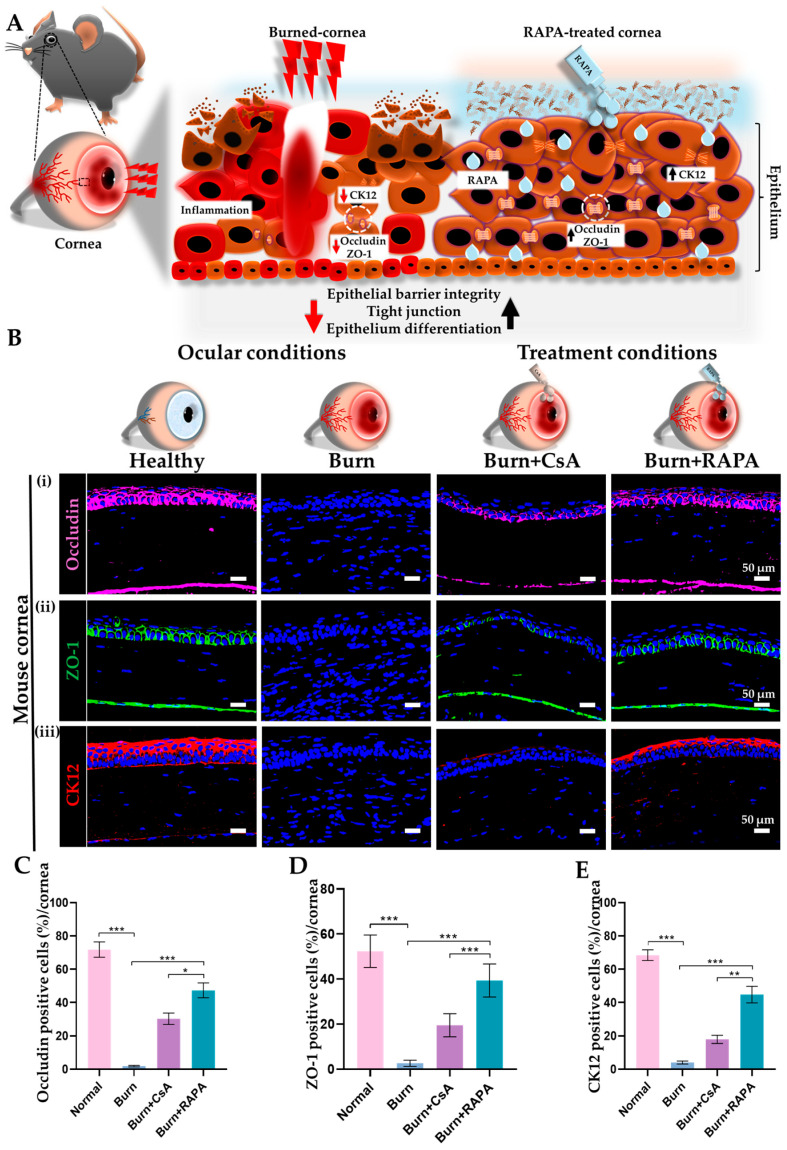
RAPA preserves the corneal epithelial barrier integrity and maintains normal epithelial differentiation in an alkali burn mouse model. (**A**) Schematic illustration of epithelial barrier integrity (occludin), tight junctions (ZO-1), and epithelial differentiation (CK12) in the alkali-burned cornea before and after RAPA treatment. Alkali burn markedly reduces occludin, ZO-1, and CK12 expression, resulting in compromised barrier function and impaired epithelial maturation/differentiation. However, RAPA treatment preserves the expression of occludin and ZO-1, as well as CK12, thereby supporting epithelial repair, tight junction restoration, and promoting corneal wound healing. In (**A**), the white circles indicate the localization of occludin and ZO-1 in the corneal epithelium. (**B**) Immunofluorescence staining for (**i**) occludin, (**ii**) ZO-1, and (**iii**) CK12 in the cornea. Nuclei were counterstained with DAPI for visualization. Quantification of (**C**) occludin, (**D**) ZO-1, and (**E**) CK12-positive cells in the cornea. Immunopositivity in the corneal tissue was quantified relative to the total number of DAPI-positive cells. Mean ± SEM (*n* = 7). One-way ANOVA followed by Tukey’s post hoc test. * *p*  <  0.05; ** *p*  <  0.01; *** *p*  <  0.001.

Conversely, RAPA treatment resulted in a pronounced upregulation and better organization of occludin and ZO-1, along with stronger CK12 expression, compared with CsA treatment, suggesting superior preservation of epithelial barrier integrity and differentiation. Healthy corneas maintained intact tight junction architecture and robust CK12 expression (Figure 3B(i–iii),C–E).

### 2.6. Effects of RAPA on Corneal Fibrotic Tissue Deposition

Corneal stromal fibrosis was evaluated by Masson’s trichrome (MT) staining in the healthy, burn, burn + CsA, and burn + RAPA-treated groups. Among the burned groups, RAPA treatment resulted in the lowest stromal fibrotic deposition and better preservation of the normal parallel lamellar architecture compared with CsA treatment, indicating a stronger anti-fibrotic effect.

**Figure 4:**
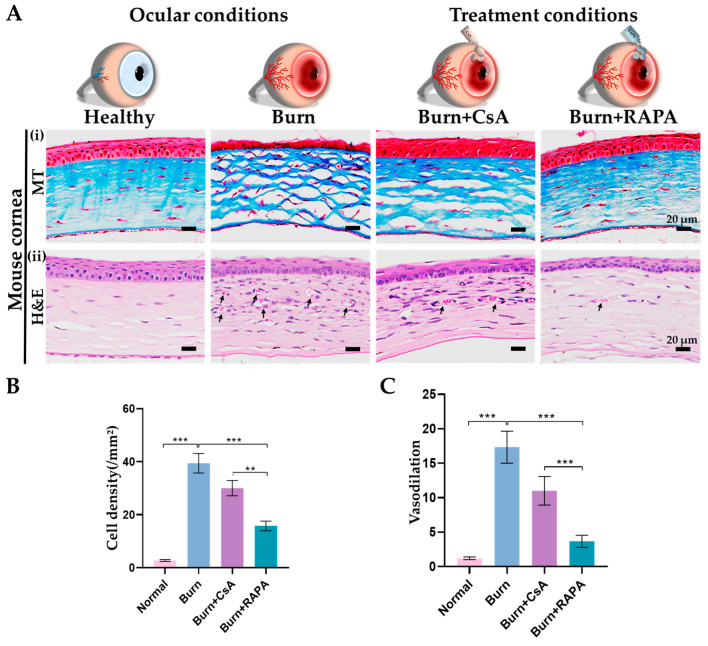
RAPA attenuates corneal stromal fibrosis, reduces inflammatory cell infiltration, and limits vasodilation in an alkali burn mouse model. (**A**) (**i**) Masson’s trichrome (MT) staining, (**ii**) Hematoxylin and Eosin (H&E) staining in the cornea. (**B**) Quantification of inflammatory cells in the corneal stroma. (**C**) Quantification of dilated blood vessels in the corneal stroma. In (**A**) (**ii**), the black arrowheads indicate the dilated blood vessels in the corneal stroma. Mean ± SEM (*n* = 7). One-way ANOVA followed by Tukey’s post hoc test. ** *p*  <  0.01; *** *p*  <  0.001.

Relative to this, the untreated burn group exhibited extensive stromal scarring and marked disruption of lamellar organization, reflecting severe fibrotic remodeling following an alkali burn. Healthy corneas showed no evidence of fibrotic alteration (Figure 4A(i)).

### 2.7. Effects of RAPA on Corneal Inflammatory Cell Infiltration and Vasodilation

Likewise, Hematoxylin and Eosin (H&E) staining was performed to evaluate inflammatory cell infiltration and vasodilation in the healthy, burn, burn + CsA, and burn + RAPA-treated groups. We observed that the burn group showed pronounced stromal infiltration of inflammatory cells along with marked vasodilation.

Surprisingly, the burn + RAPA-treated group markedly reduced infiltration of inflammatory cells and corneal vessel dilation compared with burn + CsA-treated corneas (Figure 4A(ii),B,C).

### 2.8. Effects of RAPA on Corneal Macrophage and Neutrophil Infiltration

Immune cell infiltration was assessed by myeloperoxidase (MPO) immunostaining for neutrophils and F4/80 immunostaining for macrophages in the healthy, burn, burn + CsA, and burn + RAPA-treated groups.

**Figure 5:**
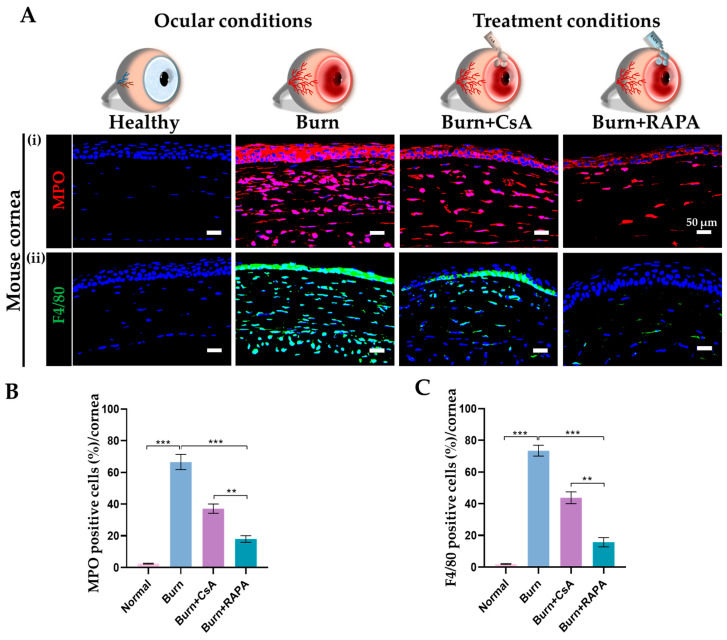
RAPA suppresses corneal immune cell infiltration in an alkali burn mouse model. (**A**) Immunofluorescence staining for (**i**) Myeloperoxidase (MPO) and (**ii**) F4/80 in the cornea. Nuclei were counterstained with DAPI for visualization. Quantification of (**B**) MPO and (**C**) F4/80-positive cells in the cornea. Immunopositivity in the corneal tissue was quantified relative to the total number of DAPI-positive cells. Mean ± SEM (*n* = 7). One-way ANOVA followed by Tukey’s post hoc test. ** *p*  <  0.01; *** *p*  <  0.001.

Notably, RAPA-treated corneas demonstrated reduced infiltration of MPO-positive neutrophils and F4/80-positive macrophages compared with CsA-treated corneas, indicating a more pronounced anti-inflammatory effect. The burn group showed robust infiltration of both immune cell populations, consistent with a strong inflammatory response following an alkali burn. The healthy group displayed no observable macrophage or neutrophil presence, confirming normal corneal immune homeostasis (Figure 5A(i,ii),B,C).

### 2.9. Effects of RAPA on Corneal Pro-Inflammatory Cytokines

**Figure 6:**
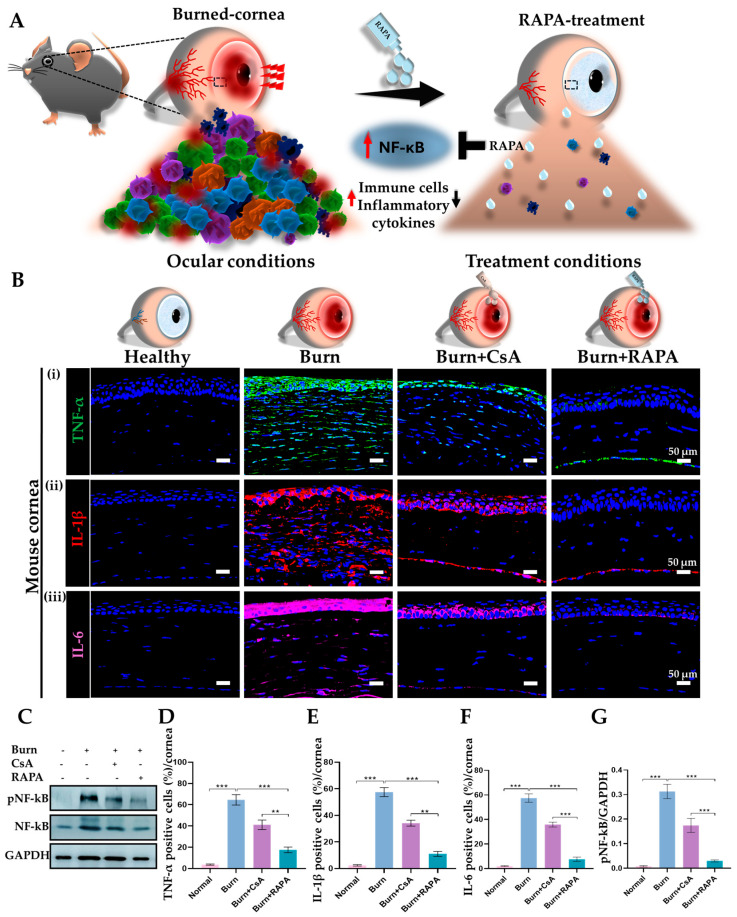
RAPA downregulates corneal pro-inflammatory cytokine expression in an alkali burn mouse model. (**A**) Graphical depiction of inflammation-associated changes in the cornea following an alkali burn and their modulation by RAPA treatment. Alkali burn induces the accumulation of inflammatory cells, upregulation of pro-inflammatory cytokines, and activation of the NF-κB signaling pathway. In contrast, RAPA treatment attenuates these inflammatory responses, highlighting its immunomodulatory role in restoring corneal immune homeostasis. (**B**) Immunofluorescence staining for (**i**) TNF-α, (**ii**) IL-1β, and (**iii**) IL-6 in the cornea. Nuclei were counterstained with DAPI for visualization. (**C**) Western blot analysis for NF-κB in the cornea. Quantification of (**D**) TNF-α, (**E**) IL-1β, and (**F**) IL-6-positive cells in the cornea. (**G**) Relative expression of NF-κB normalized to GAPDH. Immunopositivity in the corneal tissue was quantified relative to the total number of DAPI-positive cells. Mean ± SEM (*n* = 7, *n* = 3). One-way ANOVA followed by Tukey’s post hoc test. ** *p*  <  0.01; *** *p*  <  0.001.

The expression of key pro-inflammatory cytokines and mediators was evaluated in the healthy, burn, burn + CsA, and burn + RAPA-treated groups. Immunofluorescence analysis revealed markedly elevated levels of IL-1β, TNF-α, and IL-6 in the burn group, indicating robust inflammatory activation following an alkali burn. Surprisingly, RAPA treatment significantly suppressed IL-1β, TNF-α, and IL-6 expression compared with the burn group and CsA-treated group (Figure 6B(i–iii),D–F).

Likewise, Western blot analysis was conducted to evaluate NF-κB activation. NF-κB protein expression was markedly increased in the burn group, while the RAPA-treated group demonstrated significantly stronger suppression of NF-κB signaling compared with CsA treatment (Figure 6C,G).

### 2.10. Effects of RAPA on Corneal Fibrosis, Collagen Deposition, and Matrix Remodeling

**Figure 7:**
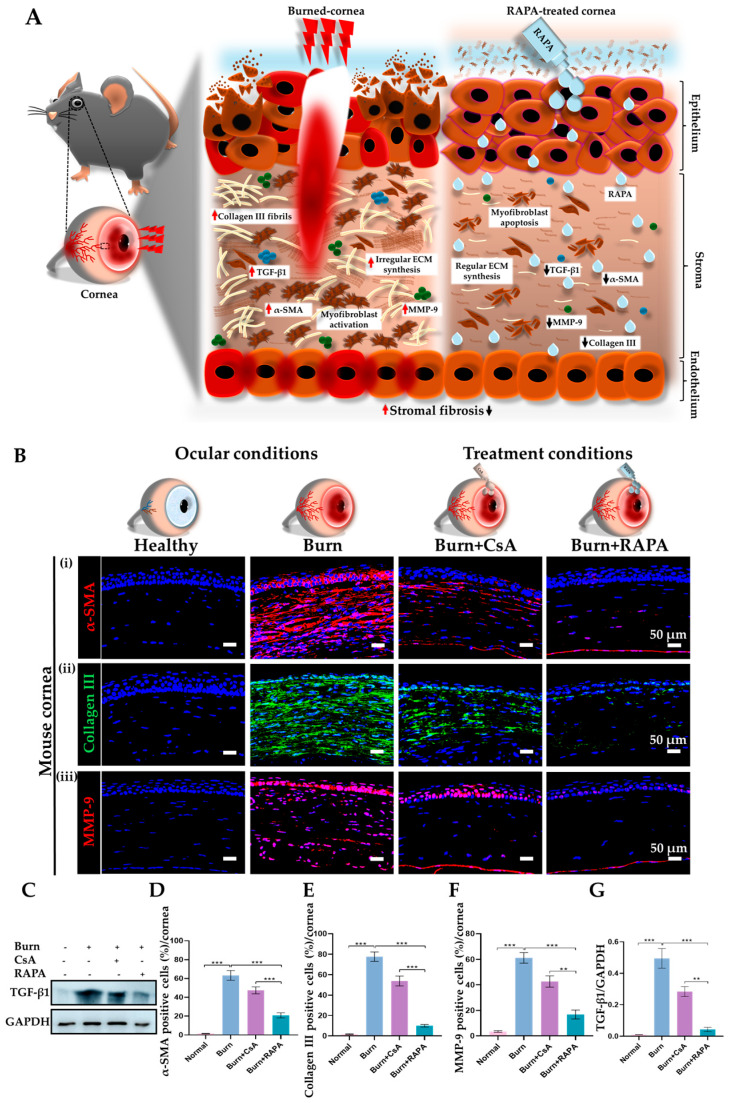
RAPA mitigates corneal stromal fibrosis in an alkali burn mouse model. (**A**) Schematic depicting corneal stromal fibrosis and its modulation by RAPA treatment in an alkali burn mouse model. Following an alkali burn, stromal fibroblasts are activated into α-SMA–positive myofibroblasts, driving excessive deposition of collagen III and upregulation of MMP-9, resulting in pathological ECM remodeling, while transforming growth factor-beta 1 (TGF-β1) acts as a central pro-fibrotic mediator orchestrating these processes. Alternatively, RAPA treatment effectively downregulates TGF-β1, suppresses myofibroblast activation, limits collagen III accumulation, and reduces MMP-9–mediated pathological matrix remodeling, collectively restoring corneal stromal architecture and preventing excessive fibrosis. (**B**) Immunofluorescence staining for (**i**) α-SMA, (**ii**) collagen III, and (**iii**) MMP-9 in the cornea. Nuclei were counterstained with DAPI for visualization. (**C**) Western blot analysis of TGF-β1 in the cornea. Quantification of (**D**) α-SMA, (**E**) collagen III, and (**F**) MMP-9-positive cells in the cornea. (**G**) Relative expression of TGF-β1 normalized to GAPDH. Immunopositivity in the corneal tissue was quantified relative to the total number of DAPI-positive cells. Mean ± SEM (*n* = 7, *n* = 3). One-way ANOVA followed by Tukey’s post hoc test. ** *p*  <  0.01; *** *p*  <  0.001.

Immunofluorescence analysis showed marked α-SMA expression, increased collagen III deposition, and elevated MMP-9 levels in the burn group. Notably, RAPA treatment significantly reduced α-SMA, collagen III, and MMP-9 expression compared with CsA treatment, indicating enhanced anti-fibrotic and matrix-stabilizing effects. Moreover, no α-SMA, collagen III, or MMP-9 expression was observed in healthy corneas, consistent with intact corneal architecture (Figure 7B(i–iii),D–F).

Similarly, Western blot analysis revealed a pronounced increase in transforming growth factor-beta 1 (TGF-β1) protein expression in the burn group. In comparison, TGF-β1 levels were dramatically reduced in the RAPA-treated group in comparison with the CsA-treated group. Notably, no detectable TGF-β1 protein was observed in healthy corneas (Figure 7C,G).

### 2.11. Effects of RAPA on Angiogenic Mediators and Endothelial Markers

Alkali burn induced pronounced corneal neovascularization, as evidenced by markedly elevated VEGF expression, increased angiopoietin-2 (Ang-2) levels, and a dense cluster of differentiation 31 (CD31)-positive vascular networks in the burn group.

**Figure 8:**
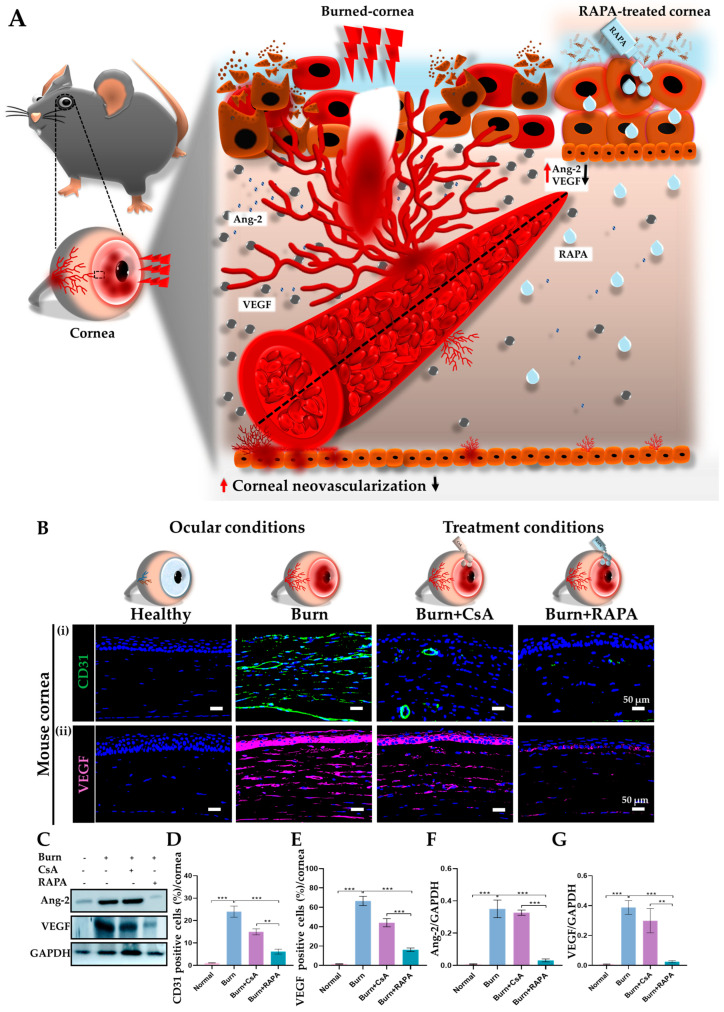
RAPA limits corneal angiogenesis in an alkali burn mouse model. (**A**) Illustrative summary of angiogenic changes induced by an alkali burn and their modulation following RAPA treatment. Following an alkali burn, VEGF is upregulated, driving pathological neovascularization, and angiopoietin-2 (Ang-2) promotes vessel destabilization and sprouting. Conversely, RAPA treatment reduces VEGF expression and downregulates Ang-2, collectively suppressing abnormal corneal neovascularization and preserving corneal transparency. (**B**) Immunofluorescence staining for (**i**) CD31, and (**ii**) VEGF in the cornea. Nuclei were counterstained with DAPI for visualization. Western blot analysis of (**C**) Ang-2 and VEGF in the cornea. Quantification of (**D**) CD31 and (**E**) VEGF-positive cells in the cornea. Relative expression of (**F**) Ang-2 and (**G**) VEGF normalized to GAPDH. Immunopositivity in the corneal tissue was quantified relative to the total number of DAPI-positive cells. Mean ± SEM (*n* = 7, *n* = 3). One-way ANOVA followed by Tukey’s post hoc test. ** *p*  <  0.01; *** *p*  <  0.001.

Distinctly, RAPA treatment significantly suppressed VEGF expression and reduced CD31-positive vessel formation compared with CsA treatment, demonstrating superior anti-angiogenic efficacy. In healthy corneas, VEGF, Ang-2, and CD31 expression were not observed, reflecting normal vascular quiescence (Figure 8B(i,ii),D,E).

Similarly, Western blot analysis demonstrated markedly elevated Ang-2 and VEGF protein expression in the burn group. The RAPA-treated group showed significantly lower Ang-2 and VEGF protein levels compared with the CsA-treated group. Ang-2 and VEGF proteins were not detected in healthy corneas (Figure 8C,F,G).

### 2.12. Effects of RAPA on Cellular Apoptosis and Dysregulated Proliferation

**Figure 9:**
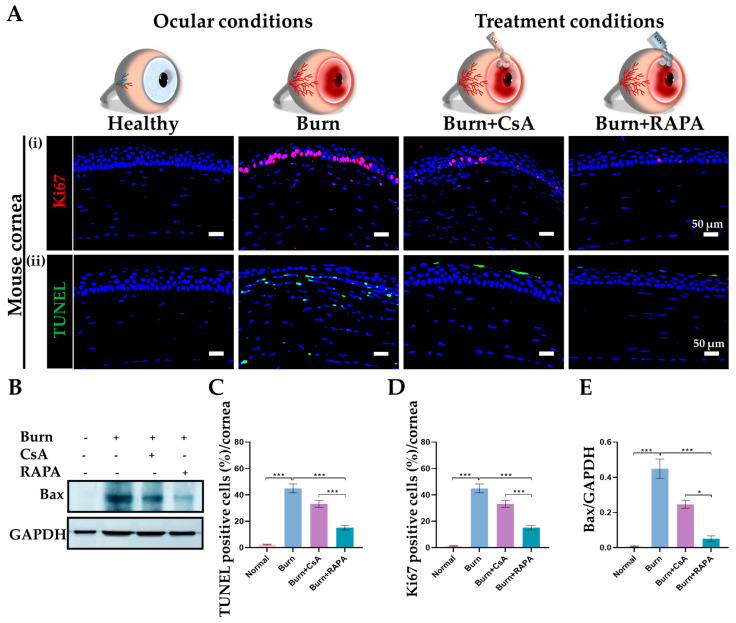
RAPA regulates corneal cellular apoptosis and proliferation in an alkali burn mouse model. (**A**) (**i**) TUNEL staining and (**ii**) Immunofluorescence staining for Ki67 in the cornea. (**B**) Western blot analysis of Bax in the cornea. Quantification of (**C**) TUNEL, (**D**) Ki67-positive cells in the cornea. Nuclei were counterstained with DAPI for visualization. (**E**) Relative expression of Bax normalized to GAPDH. Immunopositivity in the corneal tissue was quantified relative to the total number of DAPI-positive cells. Mean ± SEM (*n* = 7, *n* = 3). One-way ANOVA followed by Tukey’s post hoc test. * *p*  <  0.05; *** *p*  <  0.001.

Following an alkali burn, the cornea exhibited extensive TUNEL-positive apoptosis and disrupted Ki67 expression in the burn group, reflecting increased cell death and dysregulated proliferation. TUNEL-positive cells in the burn group were broadly distributed throughout the corneal tissue rather than being restricted to the apical epithelial layer, consistent with widespread epithelial damage. RAPA administration significantly reduced apoptotic cell numbers and Ki67 expression compared with CsA treatment, indicating improved corneal cellular viability. No detectable apoptosis or Ki67 expression was observed in healthy corneas, consistent with normal corneal cellular homeostasis (Figure 9A(i,ii),C,D).

Western blot analysis demonstrated a marked upregulation of Bax expression in the burn group. Bax protein levels were significantly lower in the RAPA–treated group than in the CsA–treated group. In contrast, the Bax protein was not detected in healthy corneas (Figure 9B,E).

## 3. Discussion

To the best of our knowledge, this is the first study to directly compare the therapeutic potential of RAPA and CsA in promoting wound healing in a mouse model of corneal alkali burn. We believe our findings represent a milestone in understanding corneal wound healing, providing a comprehensive characterization of the corneal wound and treatment responses, offering valuable insights that may reflect similar pathophysiological mechanisms and therapeutic effects in humans.

In our study, RAPA treatment demonstrated multiple beneficial effects in a mouse model of corneal alkali burn, demonstrating superiority over CsA. Specifically, RAPA treatment (1) enhanced ocular surface integrity and reduced fluorescein staining; (2) restored epithelial barrier and differentiation function; (3) preserved tear production and increased surface mucin expression; (4) suppressed corneal neovascularization; (5) diminished immune cell infiltration and downregulated pro-inflammatory cytokines; (6) attenuated stromal fibrosis; (7) reduced apoptosis and decreased dysregulated epithelial proliferation.

Interestingly, RAPA has been reported to delay wound healing in highly proliferative and vascularized tissues such as skin and lung, largely due to inhibition of mTOR-mediated cellular proliferation and regenerative signaling [24,25]. However, corneal wound repair is uniquely regulated because the cornea is an avascular and immune-privileged tissue in which excessive inflammation, fibrosis, and neovascularization are primary drivers of impaired healing and loss of transparency. In this context, suppression of pathological inflammatory and angiogenic responses may be more critical than rapid cellular proliferation [26,27,28]. Therefore, the potent anti-inflammatory, anti-fibrotic, and anti-angiogenic properties of RAPA may outweigh its anti-proliferative effects, facilitating restoration of corneal structural and functional integrity [14,15,29]. These observations highlight the tissue-specific biological effects of mTOR inhibition and further support the therapeutic relevance of RAPA in ocular surface burn.

Mechanistically, our data reinforce and extend prior evidence that the therapeutic benefits of RAPA are tightly linked to its inhibition of the mTOR axis [30]. Previous study has shown that mTOR blockade suppresses IκB kinase activity, thereby preventing NF-κB nuclear translocation and downstream cytokine induction [31]. Our results are fully consistent with this framework, as RAPA treatment markedly attenuated NF-κB activation in an alkali-burned corneas and promoted superior wound healing reflected in improved structural and functional outcomes [14,15]. Our findings not only corroborate the established literature but also provide direct experimental confirmation of RAPA’s capacity to disrupt the mTOR–NF-κB inflammatory circuit in the context of the ocular surface injury [32].

In contrast, CsA principally targets the calcineurin-nuclear factor of activated T cell (NFAT) pathway, limiting T-cell activation and cytokine release, and has been reported to exert only partial modulatory effects on NF-κB signaling [33,34]. Consistent with these mechanistic distinctions, our data show that while CsA attenuated inflammation, RAPA achieved more robust suppression of NF-κB activation and inflammatory responses. Evidence from current and prior studies suggests that mTOR inhibition more effectively controls the inflammatory and tissue-destructive responses induced by alkali burns, highlighting RAPA as a superior therapeutic modulator of corneal wound healing compared with CsA [14,35].

Activation of the NF-κB signaling cascade is recognized as a central driver of inflammation and delayed tissue repair in corneal alkali burn models [36,37]. Injured epithelial and stromal cells, together with infiltrating leukocytes, rapidly release numerous pro-inflammatory cytokines such as TNF-α, IL-1β, IL-17A, IL-6, and mediators including NOD-like receptor family, pyrin domain-containing 3 (NLRP3), and caspase-1, which amplify inflammation, exacerbate tissue damage, and delay corneal healing [38,39,40]. In our study, RAPA markedly attenuated F4/80^+^ macrophage and MPO^+^ neutrophil infiltration, decreased pro-inflammatory cytokine levels, including TNF-α, IL-1β, and IL-6, and suppressed NF-κB activation, highlighting its central role in orchestrating corneal inflammation and facilitating wound healing (Figure 6A). These findings are consistent with the prior study in the corneal allograft model, where RAPA nano-micelle ophthalmic solution more effectively prolonged graft survival, reduced CD4^+^ and CD8^+^ T cell infiltration, and preserved corneal clarity compared with CsA [22].

Additionally, corneal neovascularization is a major consequence of alkali burn and is predominantly driven by NF-κB activation, which upregulates pro-angiogenic mediators such as VEGF, Ang-2, and endothelial markers like CD31 [37,41]. Simultaneously, Ang-2, a key mediator of vascular destabilization, is elevated, amplifying pathological neovessel growth and disrupting vascular integrity. Our study demonstrates that RAPA effectively interrupts this cascade and reduces the expression of VEGF, CD31, and Ang-2, thereby limiting neovessel formation and preserving corneal structure (Figure 8A). In contrast, CsA resulted in less pronounced reductions in these angiogenic mediators. These results are consistent with a previous study in herpetic stromal keratitis, where 0.05% RAPA more effectively inhibited corneal neovascularization than 0.5% CsA, underscoring its potent capacity to modulate NF-κB-dependent angiogenic pathways and preserve corneal integrity [21].

Likewise, RAPA proved highly effective in counteracting corneal stromal fibrosis in our study. Stromal fibrosis is a major consequence of alkali burn, driven by upregulation of TGF-β1, which activates stromal fibroblasts to differentiate into α-SMA-expressing myofibroblasts. These myofibroblasts excessively deposit ECM components such as collagen III, while elevated MMP-9 activity promotes aberrant matrix remodeling. This coordinated dysregulation disrupts stromal architecture, compromises corneal transparency, and threatens visual function [42,43,44,45].

In contrast, RAPA treatment effectively suppressed TGF-β1 associated fibrotic signaling, leading to reduced myofibroblast activation and decreased expression of α-SMA and collagen III. Moreover, RAPA restored ECM homeostasis by attenuating MMP-9 activity, thereby limiting abnormal matrix turnover and preserving stromal architecture (Figure 7A).

Taken together, these results demonstrate the broader antifibrotic effects of RAPA, including inhibition of myofibroblast differentiation, reduction in excessive ECM deposition, and regulation of matrix remodeling, highlighting its superior therapeutic potential compared with CsA in preventing post-alkali burn corneal fibrosis.

Additionally, corneal alkali burn often leads to impaired tear film stability and reduced tear production due to inflammatory damage to the ocular surface and lacrimal glands [46]. Restoration of a healthy tear film begins with adequate aqueous tear production, which maintains hydration and supports the ocular surface [47]. However, sustained tear film stability also depends on the integrity of the mucin layer, which enables tears to spread evenly and adhere to the corneal epithelium [48]. Among these mucins, membrane-associated MUC1 is particularly important because it provides epithelial lubrication, reinforces the ocular surface barrier, and anchors the tear film to the corneal surface [49]. Previous studies have demonstrated that inhibition of NF-κB can improve tear production and restore ocular surface homeostasis in models of ocular injury and dry eye diseases (DED) [50,51].

Consistent with these mechanisms, RAPA treatment markedly enhanced tear secretion and stabilized the tear film following an alkali burn, accompanied by increased MUC1 expression (Figure 2B), demonstrating greater improvement than CsA.

Critically, corneal alkali burn disrupts epithelial integrity, leading to impaired barrier function and abnormal cellular differentiation, which together exacerbate ocular surface damage [52,53]. Occludin and ZO-1 play central roles in maintaining epithelial cohesion, tight junctions, and barrier stability, whereas CK12 reflects proper corneal epithelial differentiation and maturation [54,55]. Previous studies have shown that NF-κB-mediated inflammation can compromise tight junctions and disturb epithelial maturation, barrier function, and contribute to delayed wound healing [56,57,58]. In line with this, our study demonstrates that topical RAPA effectively preserves occludin and ZO-1 expression and maintains CK12-positive epithelial cells (Figure 3A), exhibiting greater efficacy than CsA in supporting epithelial barrier integrity and normal differentiation.

Moreover, NF-κB plays a central role in mediating apoptosis following an alkali burn, and excessive cell death is associated with dysregulated proliferation, ultimately impairing effective corneal wound healing [59,60]. Prior studies have shown that modulating NF-κB and controlling inflammation can simultaneously reduce apoptosis and promote cell proliferation across various ocular disease models [61,62]. Bax, a critical pro-apoptotic protein, is often upregulated in response to inflammatory signaling in the cornea, contributing to epithelial and stromal cell death after burn. In this regard, our study reveals that RAPA exerts a dual protective role in an alkali-burned corneas, markedly decreasing apoptotic cell death, likely through downregulation of Bax, while modulating epithelial proliferation, as indicated by decreased Ki-67 expression, compared with CsA–treated corneas. Consistent with previous in vitro study in human corneal keratocytes, CsA was less effective at preventing apoptosis and supporting cell proliferation compared with other therapeutic agents [63].

In addition to mTOR–NF-κB signaling, RAPA is also known to modulate autophagy, which plays a critical role in cellular homeostasis and tissue repair following injury. Activation of autophagy has been implicated in the regulation of corneal epithelial survival and stromal remodeling under stress conditions. Furthermore, emerging evidence suggests that RAPA may influence oxidative stress responses, which are closely associated with corneal inflammation and neovascularization in ocular surface diseases [64]. Dysregulated oxidative stress has been identified as a key contributor to tissue damage and pathological angiogenesis in corneal injury models, highlighting it as an important therapeutic target [65,66]. Together, these mechanisms suggest that the beneficial effects of RAPA in corneal alkali burn may extend beyond anti-inflammatory signaling to include regulation of autophagy and oxidative stress pathways.

Although the results of our study are promising, several limitations should be acknowledged. The mouse model of corneal alkali burns, while reproducible, may not fully mimic human corneal injury and repair. Species differences in structure, immunity, and regeneration may limit direct translation. This study also focused on short-to mid-term outcomes; long-term effects of RAPA and CsA on corneal healing, vision, and safety remain unknown. In addition, the relatively severe slit-lamp appearance observed in this model reflects the controlled experimental induction of alkali injury, which is designed to ensure reproducible and standardized pathology rather than fully replicate clinical variability. Similarly, this may account for differences between experimental and clinical presentations. Further research is needed to define their molecular mechanisms, cellular targets, and potential off-target effects. Moreover, as both RAPA and CsA have demonstrated strong efficacy in corneal transplantation settings [67,68], monitoring topical combination approaches in future studies may help determine whether synergistic benefits exist. Overall, comprehensive investigations are required to optimize delivery strategies and fully harness the regenerative potential of RAPA in corneal alkali burn.

## 4. Materials and Method

### 4.1. Experimental Animals

Forty female C57BL/6 mice (8 weeks old, 20–25 g; Orient Bio, Seongnam, Republic of Korea) were included in this study. Animals were housed under standardized conditions with a 12 h light/dark cycle, controlled temperature and humidity, and unrestricted access to sterilized food and water. All experimental procedures conformed to the ethical standards of the University of Ulsan College of Medicine and received approval from the Institutional Animal Care and Use Committee (IACUC approval No. A20242479).

### 4.2. Induction of Corneal Alkali Burn Mouse Model

A corneal alkali burn mouse model was established following previously described protocols [69,70]. Mice were anesthetized with intraperitoneal injections of Zoletil^®^ (50 mg/kg; Virbac, Carros, France) and Rompun^®^ (10 mg/kg; Bayer, Seoul, Republic of Korea). A 2 mm filter paper disc soaked in 0.5 N NaOH was applied to the central cornea for 10 s. Excess alkali was removed by immediate irrigation with sterile PBS for 1 min (Figure 1A).

Following an alkali burn, mice were randomly assigned to four groups (*n* = 10 each): (1) healthy, (2) burn, (3) burn+cyclosporine A (CsA; 0.05%), and (4) burn + RAPAmycin (RAPA; 0.5 mg/mL; Sigma-Aldrich, St. Louis, MO, USA). Treatments were applied topically three times daily for 14 consecutive days. On day 15, mice were humanely euthanized via CO_2_ inhalation, and corneas were harvested for histological, immunohistochemical, and protein analyses.

### 4.3. Corneal Clinical Score

Corneal damage was evaluated based on a standardized grading system [71]. The scoring criteria were as follows: 0, completely clear; 1, mild haze with visible pupil; 2, moderate opacity with partially visible pupil; 3, dense opacity with barely visible pupil; 4, total opacity obscuring the pupil.

### 4.4. Tear Secretion Measurement

Tear production was assessed using the Zone-Quick phenol red thread test as previously reported [61]. Threads were placed in the lateral lower conjunctival fornix for 15 s, and the moistened length was measured with a digital caliper (Monos, Seoul, Republic of Korea) (Figure 2A). Measurements were performed on days 1, 7, and 14 post-injuries to monitor changes over time.

### 4.5. Corneal Fluorescein Staining

Corneal epithelial integrity was evaluated with 1% fluorescein sodium (Sigma-Aldrich, Darmstadt, Germany). After 2 min of exposure, excess dye was washed off with artificial tears. Corneas were examined under cobalt blue illumination, and images were captured for analysis. The extent of the corneal epithelial defect was measured.

### 4.6. Corneal Neovascularization Grading

Corneal neovascularization was evaluated according to the previous protocol [72]. Briefly, grade 0, no new vessels; grade 1, mild neovascularization from the limbus; grade 2, moderate vessels extending toward the corneal center; grade 3, severe vessels reaching or crossing the central cornea.

### 4.7. Histological Analysis

Corneal tissues were paraffin-embedded and sectioned at 5 μm. Sections were deparaffinized, rehydrated, and stained with hematoxylin and eosin (H&E; Abcam, Cambridge, UK) or Masson’s Trichrome (MT; Sigma, St. Louis, MO, USA). Slides were mounted and examined under a light microscope to evaluate tissue morphology, fibrosis, vasodilation, and cellular infiltration.

### 4.8. Immunofluorescence Staining

The tissue sections were deparaffinized, rehydrated, and incubated with primary antibodies targeting: ZO-1 (ab617300; Abcam, 1:200), occludin (91131; Cell Signaling Technology, Danvers, MA, USA, 1:100), F4/80 (ab6640; Abcam, 1:200), MPO (ab23910; Abcam, 1:200), IL-1β (ab315084; Abcam, 1:100), Ki67 (ab15580; Abcam, 1:200), VEGF (MA1-16629; Invitrogen, Carlsbad, CA, USA, 1:100), α-SMA (ab5694; Abcam, 1:100) CD31 (ab230718; Abcam, 1:100), MMP-9 (ab73734; Abcam, 1:200), MUC1(ab45167; Abcam, 1:500), CK12 (PA5-71744; Invitrogen, 1:100), IL-6 (ab9324; Abcam, 1:200), TNF-α (3707; Cell Signaling Technology, 1:100), collagen III (ab6310; Abcam, 1:200). After 24 h of incubation, sections were washed with PBS and subsequently incubated in the dark at room temperature for 1 h with secondary antibodies conjugated to Alexa Fluor 488 (A11008; Invitrogen, 1:400), Alexa Fluor 555 (A21424; Invitrogen, 1:400), Alexa Fluor 568 (A10042; Invitrogen, 1:400), Alexa Fluor 647 (A21469; Invitrogen, 1:400), or Alexa Fluor 488 (A11029; Invitrogen, 1:400). Nuclei were counterstained with DAPI for 10 min, and the sections were subsequently mounted. Images were acquired using a confocal microscope (Carl Zeiss, Jena, Germany) and quantified using ImageJ software (version 1.62f).

### 4.9. Western Blotting

Corneal tissues were homogenized in ice-cold RIPA lysis buffer supplemented with protease inhibitors, and total protein was extracted for analysis. Equal amounts of protein were separated by SDS–PAGE and transferred onto PVDF membranes. The membranes were then blocked and incubated with primary antibodies against NF-κB (8242S; Cell Signaling Technology, 1:1000), pNF-κB (3033S; Cell Signaling Technology, 1:1000), VEGF (MA1-16629; Invitrogen,1:100), Ang-2 (2948; Cell Signaling Technology, 1:1000), TGF-β1 (ab17087; Abcam, 1:1000), Bax (sc-20067; Santa Cruz Biotechnology, Dallas, TX, USA, 1:1000), and GAPDH (2118S; Cell Signaling Technology, 1:10,000). The membranes were subsequently exposed to HRP–conjugated secondary antibodies, and the immunoreactive signals were developed using an enhanced chemiluminescence detection system (WBKLS0100; MilliporeSigma, Burlington, VT, USA).

### 4.10. TUNEL

Apoptotic cells were detected using the TUNEL assay kit (Roche, Penzberg, Germany) following the manufacturer’s instructions. Corneal sections were processed, and nuclei were counterstained with DAPI. Fluorescent images were acquired using a confocal microscope (Carl Zeiss, Jena, Germany).

### 4.11. Statistical Analysis

Statistical analyses were performed using GraphPad Prism (version 5.01, GraphPad Software), and image quantification was conducted using ImageJ (version 1.62f). Data are expressed as mean ± the standard error of the mean (SEM). One-way ANOVA followed by Tukey’s test was used to assess the effects of multiple treatments. Bartlett’s test was applied to assess variance in the in vivo experiments. Statistical significance was defined as *p* < 0.05.

## 5. Conclusions

In conclusion, RAPA significantly improves corneal wound healing in a mouse model of alkali burn by modulating NF-κB activity and enhancing key structural and functional outcomes compared with CsA. These findings highlight RAPA’s strong therapeutic promise in preclinical models and support its potential applicability in human patients, reinforcing the development of RAPA as a treatment option for severe corneal burns.

## Data Availability

The original contributions presented in this study are included in the article. Further inquiries can be directed to the corresponding authors.

## Abbreviations

RAPA	Rapamycin
α-SMA	α-Smooth Muscle Actin
VEGF	Vascular Endothelial Growth Factor
CsA	Cyclosporine A
mTOR	Mammalian Target of Rapamycin
PBS	Phosphate-Buffered Saline
MMP-2	Matrix Metalloproteinase-2
NaOH	Sodium Hydroxide
IL-1β	Interleukin 1-β
TNF-α	Tumor Necrosis Factor-α
IL-6	Interleukin 6
MMPs	Matrix Metalloproteinases
IL-17A	Interleukin-17A
CD31	Cluster of Differentiation 31
CD45	Cluster of Differentiation 45
MMP-9	Matrix Metalloproteinase-9
CK12	Cytokeratin-12
ECM	Extracellular Matrix
TUNEL	Terminal Deoxynucleotidyl Transferase UTP nick end labeling
NF-κB	Nuclear Factor Kappa-Light-Chain-Enhancer of Activated B Cells
TIMPs	Tissue Inhibitors of Metalloproteinases
MT	Masson’s Trichrome
NLRP3	NOD-Like Receptor Family Pyrin Domain Containing 3
H&E	Hematoxylin and Eosin
MUC1	Mucin 1
Ang-2	Angiopoietin-2
TGF-β1	Transforming Growth Factor-β1
MPO	Myeloperoxidase
